# Editorial: Insights in terrestrial microbiology: 2023/2024

**DOI:** 10.3389/fmicb.2025.1583425

**Published:** 2025-03-18

**Authors:** Paola Grenni, Ruibo Sun, Jeanette M. Norton

**Affiliations:** ^1^Water Research Institute, National Research Council (IRSA-CNR), Rome, Italy; ^2^NBFC, National Biodiversity Future Center, Università degli Studi di Palermo, Palermo, Italy; ^3^College of Resources and Environment, Anhui Agricultural University, Hefei, China; ^4^Department of Plants, Soils and Climate, Utah State University, Logan, UT, United States

**Keywords:** peatlands, biogeochemistry, methane, hydrogen, saline soil, biocrust, soil reclamation

**Figure 1:**
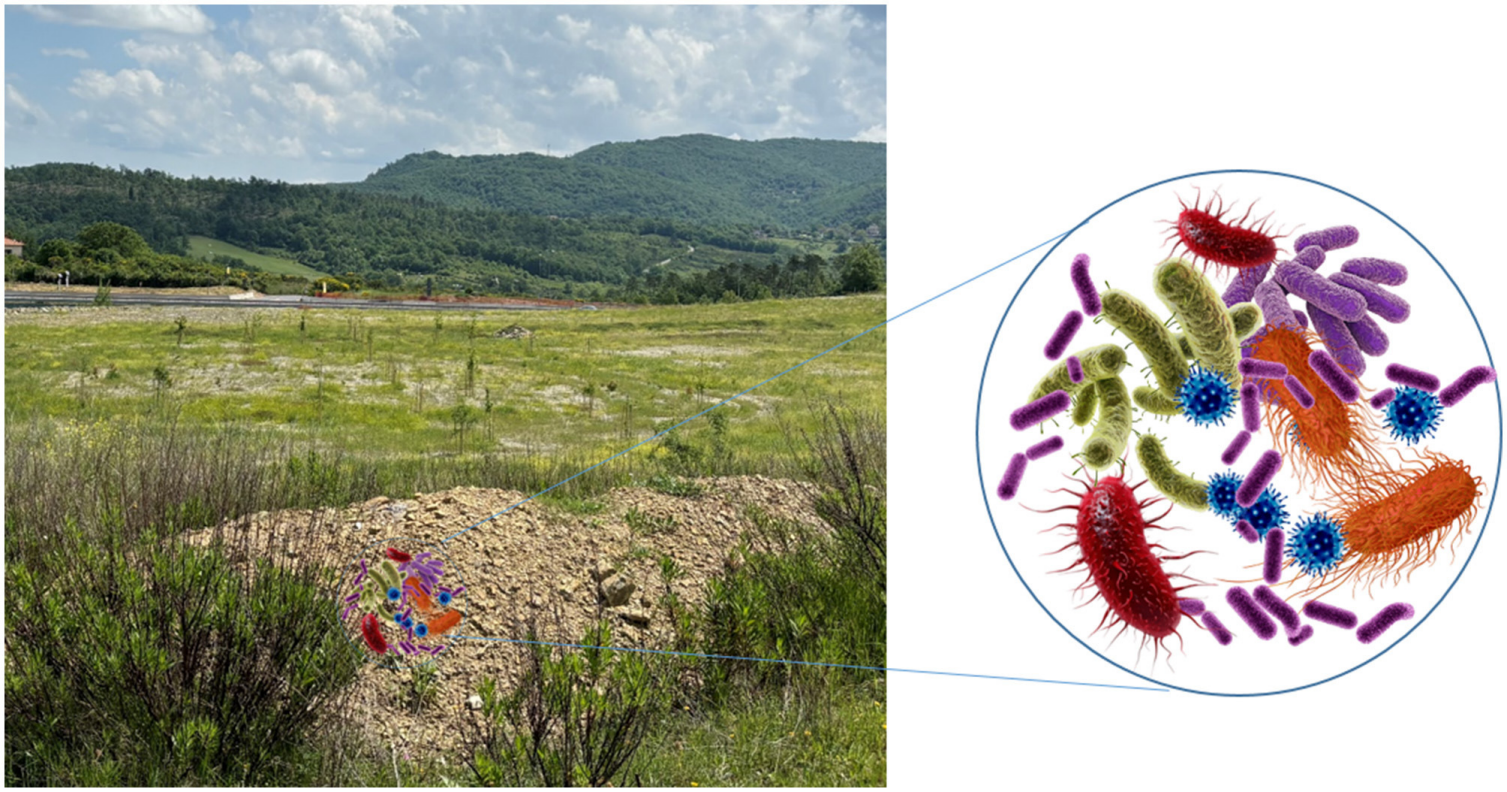
Microbial communities of soil have key roles such as: recycling nutrients, restoring soils after contamination, and climate regulation affecting the balance of CO_2_ emissions.

The role of microorganisms in terrestrial ecosystems is crucial for several functions, including recycling nutrients and restoring soils after contamination (Grenni et al., 2023; Kotschik et al., 2023; Onet et al., 2025) (Figure 1). The Terrestrial Microbiology section of Frontiers (https://www.frontiersin.org/journals/microbiology/sections/terrestrial-microbiology) aims to highlight these key roles, addressing the study and comprehension of the various microbial functions in terrestrial ecosystems. In 2025, this section has a new mission statement that emphasizes the importance of the United Nations Sustainable Development Goals (SDGs), to advance knowledge and promote innovation for contributing to global sustainability and address major environmental challenges. In fact, the section brings together contributions on microbial functions in terrestrial ecosystems, the adaptation and evolution of microbial communities, biogeochemical cycles, the structure and functioning of the soil food web, and the interactions between microorganisms and their environment, contributing insights to various United Nations Sustainable Development Goals: SDG 13 (Climate Action), SDG 14 (Life Under Water), and SDG 15 (Life on Earth).

According to these aims, the Research Topic “*Insights in terrestrial microbiology: 2023/2024*” collected nine articles that address several important aspects of the microbial community of soil ecosystems.

Soil has an essential role in maintaining carbon balance on Earth and the soil microbiome mediates carbon pools and therefore climate regulation by affecting the balance of CO_2_ emissions. This equilibrium strongly depends on the catalytic functions performed by microbial communities on soil organic matter (Yang et al.). The latter can be used by microorganisms (for their growth by anabolic reactions) or dissipated as CO_2_ to the environment by catabolic reactions. The kinetics of the organic matter and energy fluxes were studied by Yang et al. through calorimetry, and specific data processing, gaining an improved understanding of the relations between matter and energy fluxes in tightly controlled soil systems.

Kujala et al. unraveled the crucial role of the soil microbiome in the trophic interactions between fermenters and methanogens in peatlands, ecosystems which are, despite their importance, currently poorly understood. Peatlands are considered invaluable but vulnerable ecosystems where huge amounts of organic carbon are stored, with the carbon in the deep peat remaining stable due to limited thermodynamic energy and transport (Rajakaruna et al., 2024). However, peatlands emit greenhouse gases such as carbon dioxide (CO_2_) and methane (CH_4_). Methanogenesis is an anaerobic respiration that produces CH_4_ as the final product of metabolism, and it is performed by methanogens, which are strictly anaerobic Archaea (Lyu et al., 2018). The CH_4_-emitting peatland microbial community showed a pronounced response to additional substrates for fermentation and hydrogenotrophic methanogenesis, indicating high potential activity of both processes. These results indicate that the identification of active primary and secondary fermenters, the role of acetogens, the pathways for anaerobic conversion of acetate to methane, and the taxa involved are key challenges to be considered in future studies. Rolland et al. also examined the role of hydrogen consumers and methanogens in environments of long-term radioactive waste disposal. Microbial systems were observed to consume H_2_ and demonstrating the potential to contribute positively to the long-term safety of a radioactive waste repository.

The impact of continuous cropping in terms of autotoxicity, due to accumulation and imbalance of microorganisms in soil, which can lead to crop failure, and this was the focus of the study by Xing et al.. The research focuses on a long (more than 4 years) cultivation of potato, analyzing the potato root exudates and the bacterial and fungal communities around potato plants. The communities were significantly changed, with a substantial reduction of beneficial bacteria and accumulation of harmful fungi. Moreover, differentially expressed metabolites were significantly correlated with microflora biomarkers, suggesting that the continuous cultivation of potato changed the microorganism's metabolism in response to root exudates and pushed the rhizosphere microflora in a less favorable direction for crop growth. These microbial community aspects must be considered in effective crop management to guarantee potato productivity, as it is an important global staple used for food, feed, and raw materials in the industry due to its high yield, tolerance to wet environments, and great adaptability. Tea agricultural systems were investigated for their bacterial and fungal communities and potential for biocontrol of pathogens by Jibola-Shittu et al.. Further characterization of the roles and functions of the microbiomes will be required to create more sustainable tea systems.

Microbial communities in forest soils drive a variety of functions, including forest soil carbon turnover, with a key role in ecosystem services that promote forest health (Onet et al., 2025). Plant community dynamics in forest succession have been the subject of considerable attention in recent decades, but information on soil microbial communities involved in the carbon cycle is still limited. This was the main focus of Hu et al. investigating the soil microbial community composition and carbohydrate degradation potential in subtropical forests in China. Interestingly, although bacteria and fungi abundances in soil increased with forest succession in relation to both soil and litter characteristics, the diversity remained unchanged (for bacteria) or decreased (for fungi). Some important microbial functional genes related to carbohydrate degradation (e.g., cellulase, hemicellulase, and pectinase) were correlated with specific soil abiotic factors (organic carbon, total nitrogen, and moisture) and increased with forest succession, while amylase was mainly affected by soil total phosphorus and litterfall. The role of the rare biosphere was emphasized by the results of Dong et al. on the biocrust communities in karst systems. The biogeochemical transformations and enzymatic functions described are crucial to these wildland sites but also may be influential in the restoration of disturbed ecosystems.

The microbial community in sediments of sewer systems from distinct urban areas (multifunctional, commercial, and residential) was examined by Xia et al.. The overall microbial communities were related to physicochemical properties (pH and nutrients), together with the type of sewer sediment, although in-depth investigations of prokaryotic communities in sediments on a larger scale and with greater depth have to be performed to confirm these findings. The role of microbial transformations may also be instrumental in the restoration of saline alkali soils (Li et al.). Large land areas under irrigation are degraded by severe accumulation of salts, their poor physical conditions, and nutrient imbalances, including decreased available phosphorus. Technologies for improving these degraded soils require combinations of amendments, selective leaching, and biological improvements. The authors contend that combinations of phosphogypsum and phosphate solubilizing microorganisms can work toward improvements in these degraded salt-alkali soils.

The role of microbial communities in elemental cycling, soil remediation, and intensive agriculture highlights how microbial management is an important tool for progress toward achieving the UN Sustainable Development Goals for healthy soils.
